# Hypervariable Region Polymorphism of mtDNA of Recurrent Oral Ulceration in Chinese

**DOI:** 10.1371/journal.pone.0045359

**Published:** 2012-09-19

**Authors:** Mao Sun, Shan-Min Fu, Li-Feng Wang, Guang-Ying Dong, Dan Wu, Guo-Xia Wang, Yuanming Wu

**Affiliations:** 1 Center for DNA Typing, Fourth Military Medical University, Xi’an, China; 2 Department of Biochemistry and Molecular Biology, Fourth Military Medical University, Xi’an, China; 3 Department of Orthodontics, Fourth Military Medical University, Xi’an, China; 4 School of Stomatology, Fourth Military Medical University, Xi’an, China; 5 Department of Periodontics and Oral Medicine, Fourth Military Medical University, Xi’an, China; University of Southern California, United States of America

## Abstract

**Background:**

MtDNA haplogroups could have important implication for understanding of the relationship between the mutations of the mitochondrial genome and diseases. Distribution of a variety of diseases among these haplogroups showed that some of the mitochondrial haplogroups are predisposed to disease. To examine the susceptibility of mtDNA haplogroups to ROU, we sequenced the mtDNA HV1, HV2 and HV3 in Chinese ROU.

**Methodology/Principal Findings:**

MtDNA haplogroups were analyzed in the 249 cases of ROU patients and the 237 cases of healthy controls respectively by means of primer extension analysis and DNA sequencing. Haplogroups G1 and H were found significantly more abundant in ROU patients than in healthy persons, while haplogroups D5 and R showed a trend toward a higher frequency in control as compared to those in patients. The distribution of C-stretch sequences polymorphism in mtDNA HV1, HV2 and HV3 regions was found in diversity.

**Conclusions/Significance:**

For the first time, the relationship of mtDNA haplogroups and ROU in Chinese was investigated. Our results indicated that mtDNA haplogroups G1 and H might constitute a risk factor for ROU, which possibly increasing the susceptibility of ROU. Meanwhile, haplogroups D5 and R were indicated as protective factors for ROU. The polymorphisms of C-stretch sequences might being unstable and influence the mtDNA replication fidelity.

## Introduction

Recurrent oral ulceration (ROU) is one of the most common oral mucosal diseases, affecting 25% of the population [1]. Recurrent oral ulceration may sometimes be atypical in that it clinically resembles recurrent aphthous stomatitis (RAS) but neither commences in childhood, fails to resolve with age, or is associated with other features not typically associated with classical RAS, such as fever [2]–[7]. However, the cause of ROU is still unclear so far. As a complex disease with significant genetic contribution, ROU may have a complicated etiology including both genetic and environmental factors. Under some circumstances, ROU patients may have some genetic tendencies or congenitally genetic abnormalities. When some environmental factors, such as microbial infection, stimulate the body, the pathological response may alternatively lead to the disease [8].

The human mitochondrial genome (mtDNA) is a small 16,569 bp molecule of double stranded DNA. The mtDNA encodes 13 protein subunits of multimeric oxidative phosphorylation, two rRNAs and all the tRNAs required for the translation of its mRNA [9]. It has non-coding regions at displacement loop region (D-loop) that contains three hypervariable segments (HV1, HV2 and HV3) with high polymorphism [10]–[12]. Most of the mutations observed in both mtDNA coding and non-coding regions have occurred on preexisting haplogroups and define the individual mtDNA types or haplotypes [13]. Haplogroups can have important implication for understanding of the relationship between the mutations of the mitochondrial genome and disease [14], [15]. There is growing evidence that certain mtDNA clusters are associated with distinct disorders [16]. To examine the susceptibility of mtDNA haplogroups to ROU, we sequenced the mtDNA HV1, HV2 and HV3 in Chinese.

## Results

**Figure 1:**
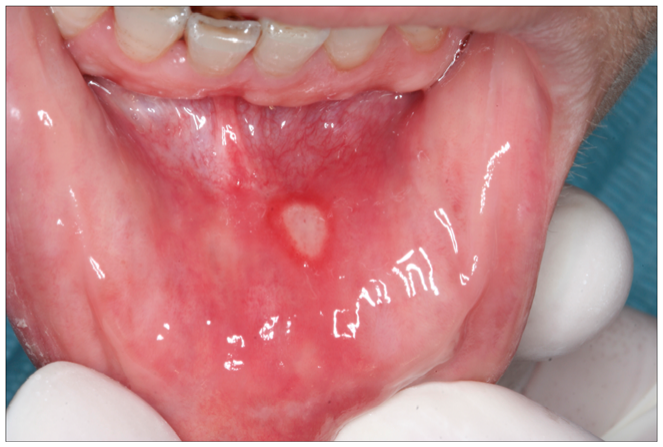
Clinical information of the patient. It is the typical symptom of ROU from a Chinese male patient. Ulcer is an oval, edge tidy and the glow around. This kind of Ulcer Belongs to the degree shallow of ROU.

**Figure 2:**
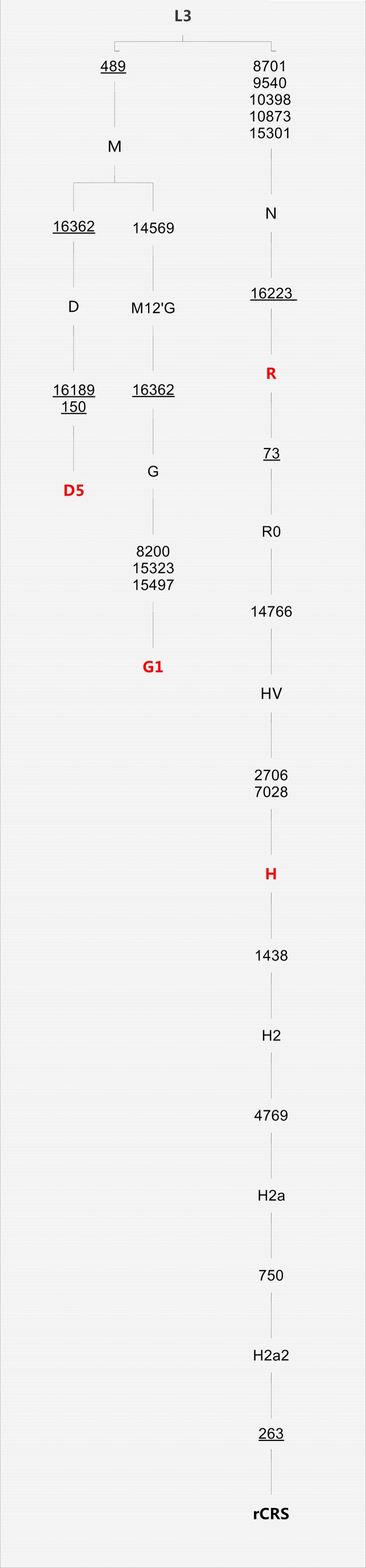
Classification tree of D5, G1, H and R complete mtDNA sequences, plus the revised rCRS. In the classification tree the red mark showed peculiar mtDNA haplogroups (the difference of them was significant in ROU patients and healthy persons.), including haplogroups D5, G1, H and R (*P*<0.05 for haplogroups D5 and G1, *P*<0.01 for haplogroups H and R).

The mtDNA haplogroups were analyzed in 249 ROU patients and 237 subjects. The typical clinical manifestations of ROU patients were shown in Fig. 1. Table 1 displayed the characteristics of the study populations, we found that the gender difference between ROU patients and healthy persons was significant (*P*<0.01). So considering the influence of gender difference, we did binary logistic regression in the data of MtDNA haplogroups and obtained the adjusted *P*-value and OR (95% CI). In Table 2, there were 16 types of haplogroups in both ROU patients and healthy persons including A, C, D, D5, F1, F, G1, H, L3, M, N, R, U5, W1, Y and Z. Twelve of them had no significance in statistics, while the other 4 types of haplogroups showed a significant difference between ROU patients and healthy persons, including D5 and G1 (P<0.05), H and R (P<0.01) (Fig. 2).

**Table 1:** Characteristics of the study populations.

	ROU patients	healthy persons	*P*-value^a^
	n = 249	n = 237	
Mean (SD) age atdiagnosis	45.4(15.2)	46.1(12.1)	
Max age at diagnosis	87	79	
Min age at diagnosis	8	10	
Male (%)	49.0	61.2	0.007

SD  =  standard deviation.

a Two test was applied chi-square test.

**Table 2:** Haplogroups frequencies and Pearson’s chi-square and OR test in ROU patients and healthy persons.

Number	Haplogroup	ROU patients	healthypersons	*P*-value^a^	Adjusted*P*-value^b^	OR	95% CI
1	A	18/249	16/237	0.836	0.963	1.017	0.502–2.057
2	C	7/249	9/237	0.542	0.592	0.758	0.276–2.086
3	D	33/249	36/237	0.541	0.583	0.866	0.518–1.448
4	D5	5/249	16/237	**0.010**	**0.025**	0.310	0.111–0.865
5	F1	12/249	20/237	0.108	0.124	0.558	0.265–1.174
6	F	5/249	4/237	1.000	0.789	1.201	0.315–4.571
7	G1	11/249	2/237	**0.015**	**0.049**	4.571	0.993–21.035
8	H	86/249	16/237	**5.513×10^−14^**	**2.336×10^−11^**	7.047	3.975–12.494
9	L3(Male)*	1/122	0/145	0.457	–	–	–
	L3(Female)*	1/127	0/92	1.000	–	–	–
10	M	44/249	48/237	0.468	0.584	0.880	0.556–1.392
11	N	10/249	11/237	0.735	0.711	0.846	0.350–2.045
12	R(Male)*	0/122	28/145	**3.046×10^−7^**	–	–	–
	R(Female)*	0/127	14/92	**5.804×10^−6^**	–	–	–
13	U5	1/249	1/237	1.000	0.958	0.928	0.056–15.237
14	W1(Male)*	0/122	0/145	-	–	–	–
	W1(Female)*	0/127	1/92	0.420	–	–	–
15	Y	1/249	4/237	0.340	0.201	0.236	0.026–2.154
16	Z	14/249	11/237	0.625	0.649	1.209	0.534–2.736

a Two test was applied chi-square test.

b Adjusted P-value: adjustment of P-values was carried out with binary logistic regression; OR (95% CI).

* Stratification chi-square test: because the change only exist in a single group (the emergence of zero), the value of partial regression coefficient (e^β^) equal to zero. So binary logistic regression is n’t suitable, P-value is calculated by stratification chi-square test.

### The Distribution of D5 and R in ROU Patients is Lower than those in Healthy Persons

**Figure 3:**
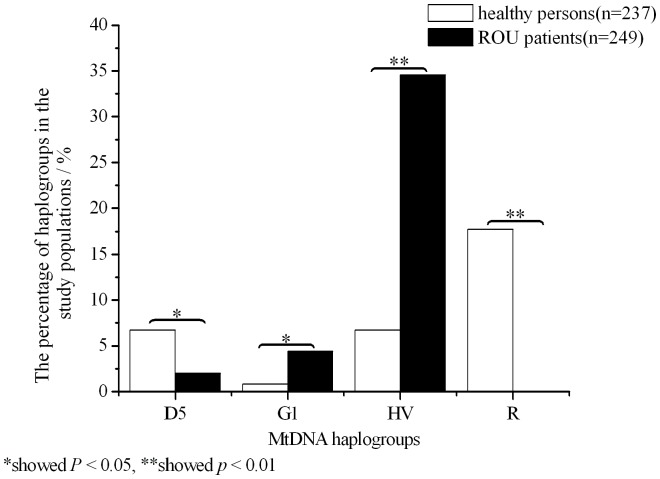
MtDNA haplogroups distribution frequency of different cohorts of ROU patients and healthy persons. X-coordinate showed four mtDNA haplogroups, y-coordinate showed the percentage of haplogroups in the study populations. * and ** respectively showed *P*<0.05 and *P*<0.01.

Haplogroup of 5 of 249 ROU patients was grouped into D5, while 16 of 237 healthy persons belonged to this type in Table 2, and the difference was significant (*P*<0.05, *OR* = 0.310). In this study, there was a certain haplogroup attracting our attention, that was R. It was not distributed in ROU patients, but haplogroup of 42 of 237 ROU patients was grouped into R. Whether male or female, the difference was much significant (Male *P* = 3.046×10^−7^ Female *P* = 5.804×10^−6^). These results showed that the percentage of D5 and R in healthy persons was significantly higher than in ROU patients. In Fig. 3 this phenomenons could be obvious: the columnar curve of D5 and R in healthy persons could be more higher than in ROU patients.

### The Distribution of G1 and H in ROU Patients is Higher than those in Healthy Persons

In Table 2, haplogroup was grouped into G1 in 11 of 249 ROU patients and 2 of 237 healthy persons (*P*<0.05, *OR* = 0.049). Haplogroup was grouped into H in 86 of 249 ROU patients and 16 of 237 healthy persons (*P* = 2.336×10^−11^, *OR* = 7.047). The distribution characteristics of haplogroup H in ROU patients and the healthy persons were similar to those of G1. The distributions showed that the percentage of G1 and H in ROU patients was significantly higher than the healthy persons respectively. In Fig. 3 the columnar curve changes of G1 and H in ROU patients significantly increased as compared with those in healthy persons.

### The Polymorphism of C-stretch in mtDNA HV1, HV2 and HV3 Regions of Healthy Persons

**Figure 4:**
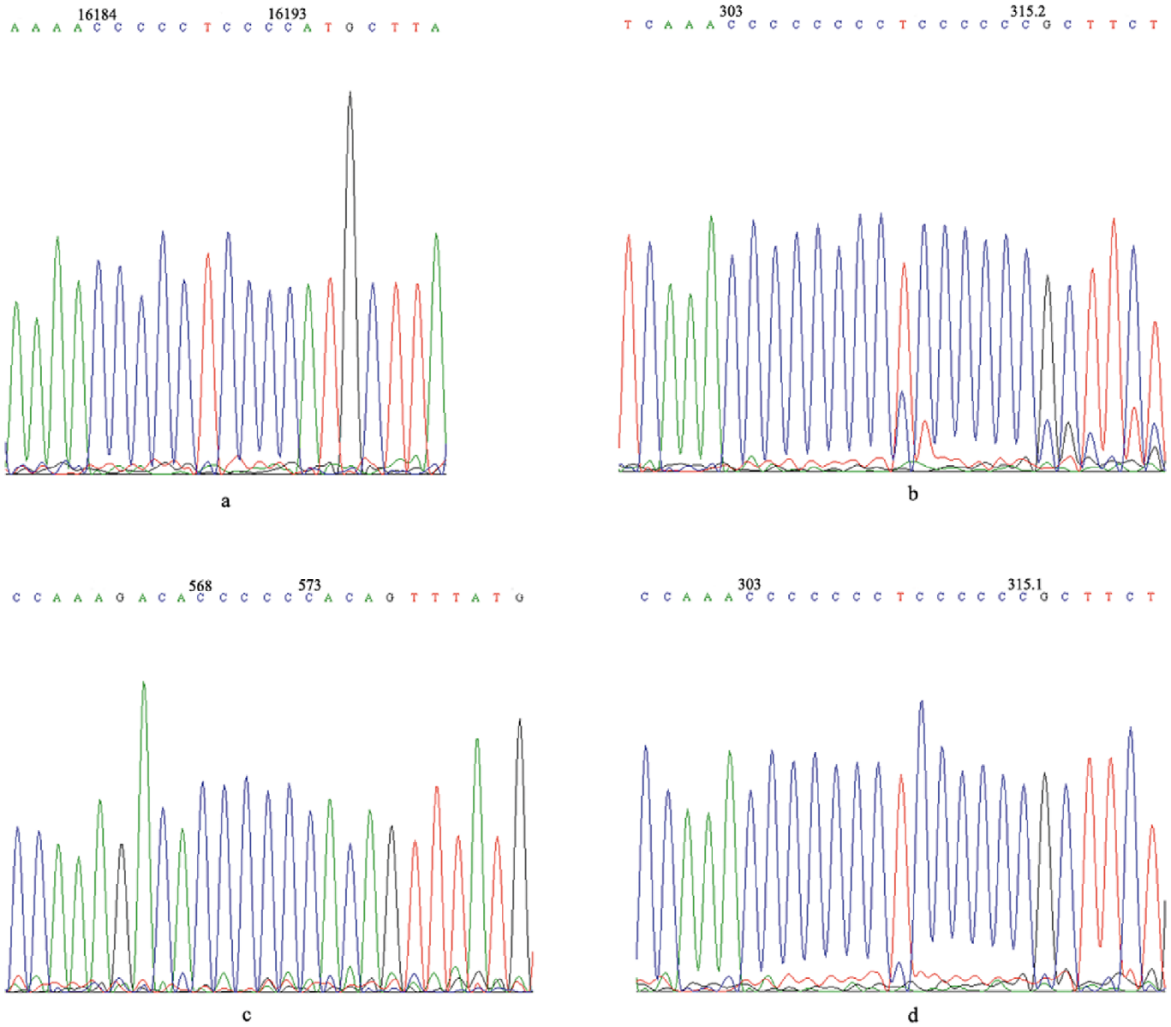
The sequences of mtDNA nt 16184–16193, nt 303–315 and nt 568–573. a. The genotype was CCCCCTCCCC in nt 16184–16193. b. The genotype was CCCCCCCCTCCCCCC in 303–315. c. The genotype was CCCCCC in nt 568–573. d. The genotype was CCCCCCCTCCCCCC in 303–315.

In nt 16184–16193, nt 303–315 and nt 568–573, there were 42 types of C-stretch sequence patterns as shown in Table 3. Of these, 65 cases were CCCCCTCCCC-CCCCCCCCTCCCCCC-CCCCCC with the highest frequency (27.43%) of C-stretch nucleotide sequence (Fig. 4a,b,c), and 1 case (0.42%) showed the same structure as the rCRS.

**Table 3:** C-stretch sequences and frequencies of mtDNA HV1, HV2 and HV3 regions in healthy persons.

	C-stretch sequences			n	%
	nt 16184–16193	nt 303–315	nt 568–573		
1	TCCCCTCCCC	CCCCCCCCTCCCCCC	CCCCCC	2	0.84
2	TCCCCTCCCC	CCCCCCCCCTCCCCCC	CCCCCC	2	0.84
3	TCCCCTCCCC	CCCCCCCTCCCCCC	CCCCCC	1	0.42
4	TCCCCCCCCC	CCCCCCCTCCCCCC	CCCCCC	1	0.42
5	CTCCCTCCCC	CCCCCCCTCCCCCC	CCCCCC	3	1.27
6	CTCCCTCCCC	CCCCCCCCTCCCCCC	CCCCCC	6	2.53
7	CTCCCTCCCC	CCCCCCCCCTCCCCCC	CCCCCC	3	1.27
8	CCTCCCCCCCC	CCCCCCCTCCCCCC	CCCCCC	1	0.42
9	CCTCCCCCCC	CCCCCCCTCCCCCC	CCCCCC	1	0.42
10	CCTCCCCCCC	CCCCCCCCTCCCCCC	CCCCCC	1	0.42
11	CCCTCTCCCC	CCCCCCCTCCCCCC	CCCCCC	1	0.42
12	CCCTCTCCCC	CCCCCCCCCTCCCCCC	CCCCCC	1	0.42
13	CCCTCCCCCCC	CCCCCCCCTCCCCCC	CCCCCC	1	0.42
14	CCCTCCCCCC	CCCCCCCTCCCCCC	CCCCCC	3	1.27
15	CCCTCCCCCC	CCCCCCCCTCCCCCC	CCCCCC	2	0.84
16	CCCTCCCCCC	CCCCCCCCCTCCCCCC	CCCCCC	1	0.42
17	CCCCTTCCCC	CCCCCCCTCCCCCC	CCCCCC	1	0.42
18	CCCCCCTCCCCC	CCCCCCCTCCCCCC	CCCCCC	1	0.42
19	CCCCCCTCCCCCC	CCCCCCCCTCCCCCC	CCCCCC	1	0.42
20	CCCCCCCCTC	CCCCCCCCTCCCCCC	CCCCCC	1	0.42
21	CCCCCCCCTC	CCCCCCCCCTCCCCCC	CCCCCC	1	0.42
22	CCCCCCCCCCCCC	CCCCCCCCCTCCCCCC	CCCCCC	2	0.84
23	CCCCCCCCCCCCC	CCCCCCCTCCCCCC	CCCCCC	2	0.84
24	CCCCCCCCCCCCC	CCCCCCCCTCCCCCC	CCCCCC	1	0.42
25	CCCCCCCCCCCC	CCCCCCCTCTCCCCCC	CCCCCC	1	0.42
26	CCCCCCCCCCCC	CCCCCCCTCCCCCC	CCCCCC	8	3.38
27	CCCCCCCCCCCC	CCCCCCCCTCCCCCC	CCCCCC	11	4.64
28	CCCCCCCCCCCC	CCCCCCCCCCCdeldel	CCCCCC	1	0.42
29	CCCCCCCCCCCC	CCCCCCCCCTCCCCCC	CCCCCC	10	4.22
30	CCCCCCCCCCC	CCCCCCCTCCCCCC	CCCCCC	10	4.22
31	CCCCCCCCCCC	CCCCCCCCTCCCCCC	CCCCCC	7	2.95
32	CCCCCCCCCCC	CCCCCCCCCTCCCCCC	CCCCCC	3	1.27
33	CCCCCCCCCC	CCCCCCCTCCCCCC	CCCCCC	3	1.27
34	CCCCCCCCCC	CCCCCCCCTCCCCCC	CCCCCC	6	2.53
35	CCCCCCCCCC	CCCCCCCCCTCCCCCC	CCCCCC	2	0.84
36	CCCCCTCCTC	CCCCCCCTCCCCCC	CCCCCC	1	0.42
37	CCCCCTCCTC	CCCCCCCCTCCCCCC	CCCCCC	3	1.27
38	CCCCCTCCCC	CCCCCCCTCCCCCCC	CCCCCC	2	0.84
39	CCCCCTCCCC	CCCCCCCTCCCCCC	CCCCCC	48	20.25
40	CCCCCTCCCC	CCCCCCCCTCCCCCC	CCCCCC	65	27.43
41	CCCCCTCCCC	CCCCCCCCCCCCC	CCCCCC	1	0.42
42	CCCCCTCCCC	CCCCCCCCCTCCCCCC	CCCCCC	15	6.33

n: No.observed, del: Nucleotide deletion, N  = 237.

### The Polymorphism of C-stretch in mtDNA HV1, HV2 and HV3 Regions of ROU Patients

The C-stretch sequence patterns for nt 16184–16193, nt 303–315 and nt 568–573 were composed of 26 types as shown in Table 4. The pattern with the highest frequency was CCCCCTCCCC-CCCCCCCCTCCCCCC-CCCCCCC with 104 cases (41.77%) (Fig. 4a,c,d) and this was followed by CCCCCTCCCC-CCCCCCCTCCCCCC-CCCCCCCC with 77 cases (30.92%) (Fig. 4a,b,c). There were no case that indicated the same structure as the rCRS.

**Table 4:** C-stretch sequences and frequencies of mtDNA HV1, HV2 and HV3 regions in ROU patients.

	C-stretch sequences			n	%
	nt 16184–16193	nt 303–315	nt 568–573		
1	CCCCCTCCCC	CCCCCCCTCCCCC	CCCCCC	1	0.40
2	TCCCCCCCCC	CCCCCCCTCCCCCC	CCCCCC	4	1.61
3	TCCCCTCCCC	CCCCCCCTCCCCCC	CCCCCC	6	2.41
4	TCCCCTCCCC	CCCCCCCCTCCCCCC	CCCCCC	1	0.40
5	CTCCCTCCCC	CCCCCCCTCCCCCC	CCCCCCC	8	3.21
6	CTCCCTCCCC	CCCCCCCTCCCCCC	CCCCCC	4	1.61
7	CTCCCTCCCC	CCCCCCCCTCCCCCC	CCCCCC	2	0.80
8	CTCCCCCCCdel	CCCCCCCCTCCCCCC	CCCCCC	1	0.40
9	CTCCCCCCCdel	CCCCCCCTCCCCCC	CCCCCC	1	0.40
10	CCTCCCCCCC	CCCCCCCCTCCCCCC	CCCCCC	2	0.80
11	CCCTCTCCCC	CCCCCCCTCCCCCC	CCCCCC	1	0.40
12	CCCTCTCCCC	CCCCCCCCTCCCCCC	CCCCCC	1	0.40
13	CCCTCCCCCC	CCCCCCCTCCCCCC	CCCCCC	2	0.80
14	CCCTCCCCCC	CCCCCCCCTCCCCCC	CCCCCC	1	0.40
15	CCCCCCCCTC	CCCCCCCTCCCCCC	CCCCCC	2	0.80
16	CCCCCTCCCT	CCCCCCCTCCCCCC	CCCCCC	1	0.40
17	CCCCCTCCCT	CCCCCCTCCCCCC	CCCCCC	3	1.20
18	CCCCCTCCCC	CCCCCCTCCCCCC	CCCCCC	1	0.40
19	CCCCCTCCCC	CCCCCCCTCCCCCCC	CCCCCC	1	0.40
20	CCCCCTCCCC	CCCCCCCTCCCCCC	CCCCCC	77	30.92
21	CCCCCTCCCC	CCCCCCCTCCCCCC	CCCCCC	4	1.61
22	CCCCCTCCCC	CCCCCCCCCTCCCCCC	CCCCCC	1	0.40
23	CCCCCTCCCC	CCCCCCCCCTCCCCCC	CCCCCCC	1	0.40
24	CCCCCTCCCC	CCCCCCCCTCCCCCC	CCCCCC	104	41.77
25	CCCCCTCCCC	CCCCCCCCTCCCCCC	CCCCCC	7	2.81
26	CCCCCTCCTC	CCCCCCCTCCCCCC	CCCCCC	12	4.82

n: No.observed, del: Nucleotide deletion, N  = 249.

## Discussion

This concerted action that the mutations of genomic DNA play an important role in the disease is complex, but mtDNA offers the possibility of approaching the problems from better defined perspective. MtDNA has already been fully sequenced [17] and many subsequent publications have revealed polymorphic sites, haplogroups and haplotypes.

Distribution of various disease groups among these haplogroups showed that some of the mitochondrial haplogroups are predisposed to disease [18]. The researchers found some haplogroups had a significantly higher occurrence in Leber's hereditary optic neuropathy (LHON) patients suggested that they are risk factors [19]–[21]. All the seven probands and their maternal relatives of Noonan syndrome (NS) were clustered under a major haplogroup R, suggesting that these haplogroups may influence NS in South Indian populations [22]. Mitochondrial haplogroup H was associated with early onset of myocardial infarction (MI) in male smokers [23]. For breast and esophageal cancer, haplogroup N was a risk factor because of mitochondrial DNA G10398A polymorphism [24]. Substitutions in the D-loop may be part of a haplotype with mutations elsewhere in the mtDNA. The hypothesis is that on their own some polymorphisms are selectively neutral, but in specific combinations they act in a synergistic, deleterious manner with established pathogenic mtDNA mutations to increase the risk of disease expression or to produce a more severe clinical outcome. The rich variability in HV1, HV2 and HV3, compared with the relatively constant constellation within the gene regions, provides useful criteria for pathogenetic studies [25].

We screened HV1, HV2 and HV3 to assess correlation between the ROU patients and the healthy persons. This was the first study to trace mtDNA H variants in ROU patients of Chinese population. Our results showed that haplogroups G1 and H were significantly more abundant in ROU patients (*P* = 0.049 for haplogroups G1 and *P* = 2.336×10^−11^ for haplogroup H) (Table 2). Thus, mtDNA haplogroups G1 and H might constitute a risk factor for ROU. These haplogroups might increase the incidence of ROU disease. On the other hand, haplogroups D5 and R were significantly more abundant in normal subjects (*P* = 0.025 for haplogroup D5, and Male *P* = 3.046×10^−7^ Female *P* = 5.804×10^−6^ for haplogroup R), suggesting that they might be protective factors for ROU.

In the process of mtDNA haplogroups to research, we found an interesting phenomenon–the generation of C-stretch. It was mainly produced by the mechanism of polymerase’s copying slippage and on the regulation of nuclear code factors to mitochondria sequences. In the process, polymerase’s copying slippage could not only produce the sequence's length polymorphism, can also cause the length heterogeneity [26]. Some scholars reported t the polymorphism of C-stretch was maternally inherited showing a similar distribution along maternal lineage and the pattern of the length heteroplasmy seems to be maintained in an individual and it was regenerated de novo by replication slippage following each cell division [27]. Along with the increase of the poly[C] number, the polymorphism of C-stretch became being unstable and influence the mtDNA replication fidelity [28].

Clinical characteristics and pathogenesis of ROU patients are rather complicated. Our results reveal association of haplogroups G1, H, D5 and R with ROU for the first time. This work may be aspiring for further studies on haplogroups in this disease, and may shed new light on the molecular pathogenesis of ROU.

## Methods

### Materials

The 249 cases of ROU patients were diagnosed by School of Stomatology Fourth Military Medical University. The diagnostic criteria [29] was shown in Table 5. Of them, 122 were male and 127 were female, aged from 8 to 87 years old. The control group included 237 healthy volunteers, of whom 145 were male and 92 were female, aged from 10 to 79 years old. The healthy persons never had a history of oral diseases.

**Table 5:** The diagnostic criteria of ROU.

	ROU patients	healthy persons
The horizontal range being covered with yellow false membrane	Yes	No
Surrounding hyperemia	Yes	No
The central sag	Yes	No
Obvious causalgia	Yes	No
The different of ictal phase	Yes	No
Cyclicality	Yes	No
self-limiting	Yes	No

All experiments were approved by the Medical Ethics Committee of the Fourth Military Medical University and the Medical Ethics Committee of the School of Stomatology of the Fourth Military Medical University. All participants provided informed written consent. Of them, 10 were the children under the age of 18 year and the guardians of the children signed informed written consent. This consent procedure was approved by the Medical Ethics Committee of the Fourth Military Medical University and the Medical Ethics Committee of the School of Stomatology of the Fourth Military Medical University.

All participants included 74 elderly people above the age of 60 year, of whom 63 were bachelor degree and fully understood the content of the consent when they signed informed written consent. Of them, 11 were other qualification and we particularly explained the content of the informed written consent to them. When they signed informed written consent, they also understood the content of the consent. A total of 74 elderly people enrolling into the study had no other diseases such as presenile dementia, Lobusparietalis, Gerstmann’syndrome and so on. This consent procedure was approved by the Medical Ethics Committee of the Fourth Military Medical University and the Medical Ethics Committee of the School of Stomatology of the Fourth Military Medical University.

All participants didn't exist in the following situation: participants who declined to participate or otherwise did not participate were eligible for treatment (if applicable) and were not disadvantaged in any other way by not participating in the study.

### DNA Extraction

0.5 mL of venous blood was dealt with EDTA-Na_2_ anticoagulation in both of the groups, and then DNA was extracted with the RelaxGene Blood DNA System of Tiangen (catalogue number: DP319–02). The quality of the extracted DNA was tested with NanoDrop 2000 (Thermo Scientific).

### Primer Design

Referencing Yao [30] et al, through the primer 6.0 software, the primer was designed. And then it’s specificity was detected via NCBI. The primer was shown in Table 6. The Beijing AUGCT DNA-SYN Biotechnology Co., LTD synthesized the primers and purified by PAGE.

**Table 6:** PCR primers.

Gene	Region	Primers
HV1	16024–16365	a: 5′CACCATTAGCACCCAAAGCT3’
		b: 5′GAGGATGGTGGTCAAGGGAC3’
HV2	73–340	a: 5′ CTCACGGGAGCTCTCCATGC 3′
		b: 5′ CTGTTAAAAGTGCATACCGCCA 3′
HV3	438–574	a: 5′ GCTTCTGGCCACAGCACTTA 3′
		b: 5′ GGTGATGTGAGCCCGTCTAA 3′

### PCR and Sequencing

PCR system was 25 µl, including 1 µ L of DNA template, 12.5 µ L of PCR Mix(100 mM Kcl, 20 mM Tris-Hcl, 3 mM Mgcl_2_, 400 µM dNTP, 0.1U/µl Taq DNA polymerases), 1 µL of 10 pmol the upstream primer, 1 µL of 10 pmol the downstream primer, the added ultrapure water to 25 µL. The research used the 2720 thermal cycler PCR instrument (AB company), circulation conditions: 95°C for 2 min followed by 35 cycles of 95°C 30 s, 60°C 30 s, 72°C 30 s, and a final extension for 5 min at 72°C. After preserved at 4°C. the 1.5% of agarose gel electrophoresis inspection was conducted, The sequence's length by the Beijing AUGCT DNA-SYN Biotechnology Co., LTD was: HV1 was 387 bp,HV2 was 321 bp and HV3 was 252 bp.

### Data Analysis

Referencing the revised Cambridge Reference Sequence (rCRS, NC_012920) [16], the sequences of HV1, HV2 and HV3 were 960 bp in all. The BioEdit software comparised the sequences, and then DNASP 5.0 software corrected them. The statistics of the polymorphic sites were conducted through Sequencher 4.1.4 software and the mtDNA haplogroups were categorized on the haplogrep (http://haplogrep.uibk.ac.at/). The experimental data were processed through SSPS 13.0 software. On the one hand, two test group (ROU patients and healthy persons) was applied chi-square test in everyone Haplogroup. The values of chi-square test were selected through difference of expected count and total number. On the other hand, in order to reduce errors we compared gender diversity between ROU patients and healthy persons. If the difference was significant, we did binary logistic regression and obtained the adjusted *P*-value and OR (95% CI).P-values below 0.05 were considered statistically significant.

Through DNA sequencing this research didn’t find new mutations in the HV1, HV2 and HV3 regions. All data were reported on the PhyloTree(http://phylotree.org/).
